# Characterization of Marine Organism Extracellular Matrix-Anchored Extracellular Vesicles and Their Biological Effect on the Alleviation of Pro-Inflammatory Cytokines

**DOI:** 10.3390/md19110592

**Published:** 2021-10-21

**Authors:** Sung-Han Jo, Seon-Hwa Kim, Changsu Kim, Sang-Hyug Park

**Affiliations:** 1 Industry 4.0 Convergence Bionics Engineering, Pukyong National University, 45 Yongso-ro, Nam-Gu, Busan 48513, Korea; josunghan91@gmail.com (S.-H.J.); seonhwakim431@gmail.com (S.-H.K.); 2 Department of Orthopedics Surgery, Kosin University Gospel Hospital, 262 Gamcheon-ro, Seo-gu, Busan 49267, Korea; mewha98@naver.com; 3 Department of Biomedical Engineering, Pukyong National University, 45 Yongso-ro, Nam-Gu, Busan 48513, Korea

**Keywords:** marine organism, extracellular matrix, extracellular vesicle, sea cucumber, anti-inflammation

## Abstract

Representative marine materials such as biopolymers and bioceramics contain bioactive properties and are applied in regenerative medicine and tissue engineering. The marine organism-derived extracellular matrix (ECM), which consists of structural and functional molecules, has been studied as a biomaterial. It has been used to reconstruct tissues and improve biological functions. However, research on marine-derived extracellular vesicles (EVs) among marine functional materials is limited. Recent studies on marine-derived EVs were limited to eco-system studies using bacteria-released EVs. We aimed to expand the range of representative marine organisms such as fish, crustaceans, and echinoderms; establish the extraction process; and study the bioactivity capability of marine EVs. Results confirmed that marine organism ECM-anchored EVs (mEVs) have a similar morphology and cargos to those of EVs in land animals. To investigate physiological effects, lipopolysaccharide (LPS)-infected macrophages were treated with EVs derived from sea cucumber, fish, and shrimp. A comparison of the expression levels of inflammatory cytokine genes revealed that all types of mEVs alleviated pro-inflammatory cytokines, although to different degrees. Among them, the sea cucumber-derived EVs showed the strongest suppression ability. This study showed that research on EVs derived from various types of marine animals can lead to the development of high value-added therapeutics from discarded marine wastes.

## 1. Introduction

Oceans are abundant sources of diverse biomaterials. Recently, researchers have focused on marine-derived biomaterials for applications in various fields, such as the biological activity of marine biomaterials, tissue engineering, and drug delivery. Various studies have been conducted on these biomaterials because of their excellent biocompatibility and diversity [1]. Among marine-derived biomaterials, the marine organism-derived extracellular matrix (ECM) is composed of structural and functional molecules [2,3,4]. The ECM possesses various beneficial cellular responses, such as promoting functional tissue reconstruction [5], angiogenesis [6], stem cell recruitment [7], and modulation of innate immune responses [8]. However, the mechanisms by which these ECMs affect cell bioactivity are not yet known. Various factors have been reported to play a role in the mechanisms underlying ECM interactions with cells, including specific surface topography, mechanical properties, cellular responses to ECM-bound integrins, and bound growth factors [9].

Extracellular vesicles (EVs) secreted from cells are divided into exosomes and microvesicles, according to their origin [10]. It is known that protein and microRNA (miRNA) cargos play an important role in cell-to-cell communication [11]. In general, mammalian EVs are found in biofluids, such as blood [12], cerebrospinal fluid [13], and urine [14]. EVs have been utilized in the field of disease diagnosis as carriers of various information cargos of proteins and genetic materials [15,16]. In addition, EVs obtained through cell culture have different abilities depending on the surrounding environment and stimulation [17]. EVs have been studied as a treatment for degenerative diseases such as stroke [18], osteoarthritis [19], and cancer [20], which are difficult to treat by treating stimulated immune cells or stem cell-derived EVs. Recent data indicate that EVs may also be exploited directly as potential therapeutic agents for tissue regeneration and immune response modulation [21].

Recent ECM studies further revealed that matrix-bound EVs can be extracted from decellularized tissue, such as the urinary bladder (UBM), small intestinal submucosa (SIS), and dermis [9,22,23]. Despite the use of a decellularizing reagent, ECM-bound EVs were not destroyed, and extraction was possible. The characterization of matrix-bound EVs revealed that they contain protein and genetic material (miRNAs) commonly involved in cell development, cellular growth, and cell cycle promotion. In addition, although there are differences in expression levels between tissues, matrix-derived EVs have tissue and organ development effects [9].

**Figure 1:**
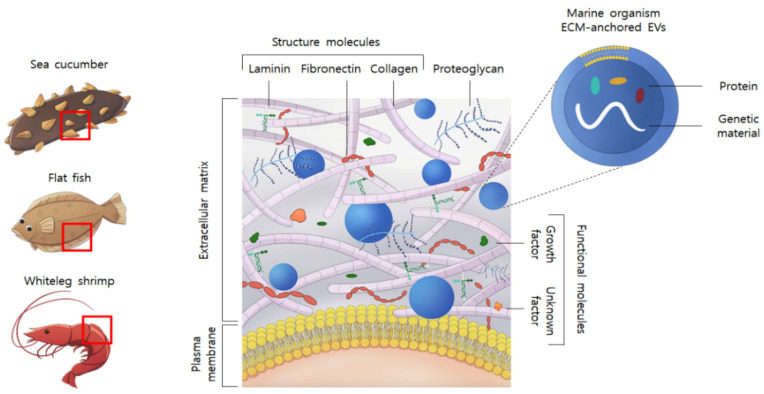
The biologically active molecular components of marine organism extracellular matrix (ECM)-anchored extracellular vesicles (EVs).

However, marine-derived EV bio-application research is still insufficient. Most of the latest research on marine EVs has been applied to the ecology and ecosystem related to EVs released from marine bacteria [24,25]. Therefore, we aimed to study marine-derived EVs that can be used in regenerative medicine. The marine ECM is composed of structural molecules, such as collagen, fibronectin, laminin, proteoglycan, and functional molecules that can act as growth factors, and it was expected that EVs would be anchored between them (Figure 1). Therefore, we decided to use the method of extracting EVs from the ECM, which was previously studied for marine organisms that are difficult to extract from pure biofluid because seawater contains bacterial EVs [24,25]. In this study, we focused on extracting EVs from marine organisms, not marine bacteria, and confirmed their biological activation. In addition, we selected fish, crustaceans, and echinoderms among representative marine organisms to confirm their applicability to a wide range (Figure 1). Here, we identified EVs embedded in the marine ECM and their anti-inflammatory effects.

## 2. Results and Discussion

### 2.1. Physical Characterization of the Marine Organism-Derived EVs

**Figure 2:**
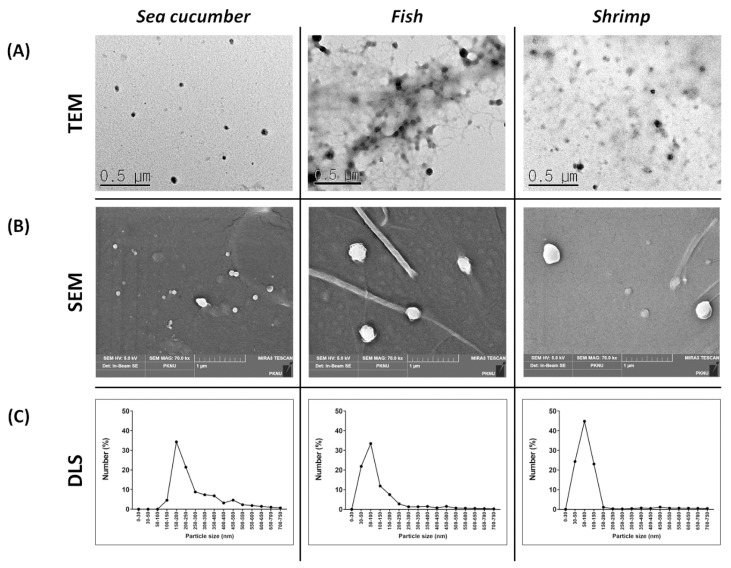
Physical characterization of marine organism ECM-anchored EVs. (**A**) Morphology of mEVs identified using transmission electron microscope (TEM). Scale bar, 0.5 μm. (**B**) Morphology of mEVs identified using scanning electron microscope (SEM). Scale bar, 1 μm. (**C**) The size distribution of each mEV was measured using dynamic light scattering (DLS). Data are presented for the average size of three independent experiments.

Various detection methods, including electron microscopy, light scattering, and molecular profiling, have been used to explore the physical properties of EVs [26,27]. In this study, we explored the physical properties of mEVs using the most commonly used methods. Marine organism ECM-derived EVs (mEVs) have not yet been studied. Therefore, several characterization methods were used to identify these mEVs. The mEVs were identified using electron microscopy and dynamic light scattering (DLS). The EVs studied to date have a membrane composed of a lipid layer and have spherical and elliptical appearances. In addition, EVs are divided into exosomes and microvesicles, with a size distribution of ≤150 and ≤1000 nm, respectively [27,28,29,30,31]. As shown in the transmission electron microscope (TEM) and scanning electron microscope (SEM) images, it was confirmed that the mEVs had a circular shape, and they appeared to have a similar shape to the general EVs studied (Figure 2A,B). Vesicles of approximately 100 nm were also observed in EM images, but the lipid bilayer observed in these vesicles in previous studies could not be confirmed (Figure 2A) [28,32]. In addition, determination of the size distribution using DLS revealed that almost all EVs were distributed below 200 nm. Although particles were detected at 400–600 nm in the sea cucumber group, they were also within the EV range (Figure 2C) [29]. These results confirmed that they have a similar appearance and size distribution to those of marine and mammalian organisms.

### 2.2. Marine Organism-Derived EVs Contain Genetic Material and Protein

**Figure 3:**
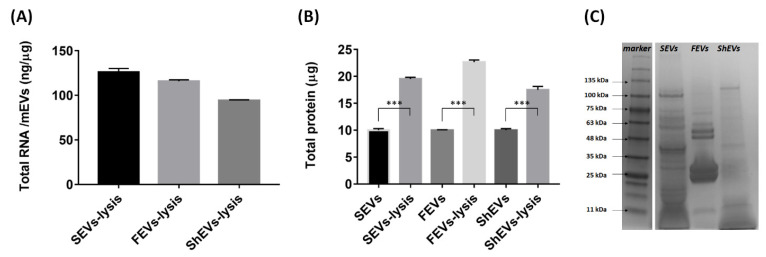
Marine organism ECM-anchored EVs internal cargo. (**A**) The amount of genetic material per mEVs protein weight was calculated based on the absorbance values measured at 260 nm. (**B**) The amount of total protein was measured using Bradford assay. (**C**) The mEVs protein cargos were compared with sea cucumber-, fish-, and shrimp-derived EVs using SDS-PAGE. Data are presented as the mean ± SD from four independent experiments. (*** *p* < 0.001 as calculated using Student’s *t*-test).

Size and morphological experiments confirmed that marine organism-derived particles have a similar shape and size to EVs, but since there is a possibility that these are ECM residues or limestone, we tried to confirm whether they are EVs by discovering genes and proteins inside the vesicles. In general, since mammalian EVs contain genes and proteins for intercellular communication and homeostasis [15,16], it was expected that mEVs would also have a similar composition. A large amount of genetic material was extracted from mEV cargo using TRIzol, and chloroform. In this study, we did not conduct experiments on the size and sequencing of the extracted genetic material (Figure 3A), but we plan to confirm this through a follow-up study. Further, we compared protein concentrations before and after destroying vesicles using cell membrane lysis buffer. When measuring the protein concentration with destroyed vesicles, sea cucumber ECM-anchored EVs (SEVs) measured 1.95 times higher than before; fish ECM-anchored EVs (FEVs) measured 2.26 times higher than before; and shrimp ECM-anchored EVs (ShEVs) measured 1.75 times higher than before (Figure 3B). In several studies, structural molecules such as collagen were mainly observed between 100 and 200 kDa in the marine ECM [33,34,35], but the mEV cargo contained proteins with various molecular weights; mainly low molecular weight proteins were observed (Figure 3C). The results confirmed that mEVs consist of a lipid layer structure that protects genetic materials and protein cargo. This packaging method is used to release materials manufactured inside the cell to the outside [36,37]. To date, it has been mainly found in terrestrial EVs.

### 2.3. Cytotoxicity Analysis

**Figure 4:**
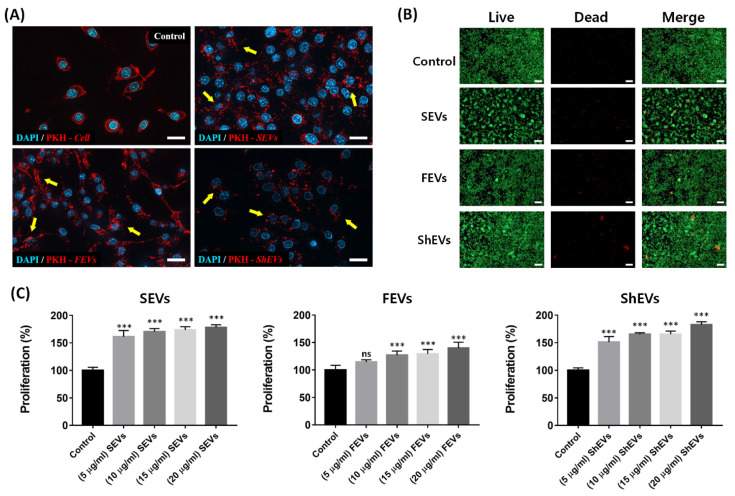
Endocytosis and cytotoxicity assay using marine organism ECM-anchored EVs. (**A**) RAW-264.7 cells were treated with PKH-26-labeled mEVs (red) for 24 h and counterstained with DAPI (blue). Upper right panel represents the cell membrane-labeled group (control); upper left panel represents the labeled SEV uptake group; left lower panel represents the labeled FEV uptake group; and right lower panel represents the labeled ShEV uptake group. Yellow arrows point out representative PKH-26-labeled mEVs. Scale bar, 20 μm. (**B**) Live/dead cell viability assay of RAW-264.7 cells cultured mEVs (20 μg/mL) suspension medium for 24 h. The live and dead cells exhibited green and red fluorescence, respectively. Scale bar, 100 μm. (**C**) The RAW-264.7 cells were treated with several concentration of mEVs (0, 5, 10, 15, 20 μg/mL) for 24 h. Cell proliferation analysis using water-soluble tetrazolium salt. Data are presented as the mean ± SD from independent experiments (*n* = 4). (*** *p* < 0.001 as calculated using one-way ANOVA).

To investigate cell endocytosis and cell toxicity, fluorescence analysis was performed. PKH-26 fluorescent dye stained the membrane of mEVs via non-covalent binding with the membrane [38]. The internalization of mEVs was detected using cell-tracking methods. Labeled mEVs were taken up into RAW 264.7 cell membrane to identify the cell response, such as a necrosis and detachment after endocytosis. In the control group, the cell membrane was directly stained, and the other groups observed the uptake of the stained mEVs. In the mEVs group, red-stained vesicles were embedded in the cell membrane, but not in the control group. The images confirmed that PKH-26-labeled mEVs could easily enter into the cell without any stimulation or cell detachment (Figure 4A). In addition, macrophages were treated with mEVs for 24 h to observe the viability changes. In SEV- and ShEV-treated cells, a large number of colonies were confirmed compared to that in the control group, and a few colonies were also observed in the FEV group. However, necrosis was not observed in any group (Figure 4B). Subsequently, the effect of mEVs on macrophage cell proliferation was examined, and the degree of mitochondrial activity was measured after treatment with various concentrations for 24 h. In the SEV group, the activity increased from 161% at 5 μg/mL to 178% at 20 μg/mL. In the FEV group, a statistically significant increase was confirmed from 114% at 10 μg/mL, and about 139% proliferation was increased at a concentration of 20 μg/mL. The activity increased in the ShEV group by 151% at 5 μg/mL and up to 182% at 20 μg/mL (Figure 4C). These results confirmed that mEVs showed no cytotoxicity and increased proliferation in a dose-dependent manner. Published descriptions of cytotoxicity and proliferation experiments conducted with land animal-derived EVs yielded similar results to those seen in the group treated with mEVs. In addition, no toxicity due to enzymes and reagents used to extract EVs from the marine-derived ECM was observed.

### 2.4. Modulation of Pro-Inflammatory Cytokine

**Figure 5:**
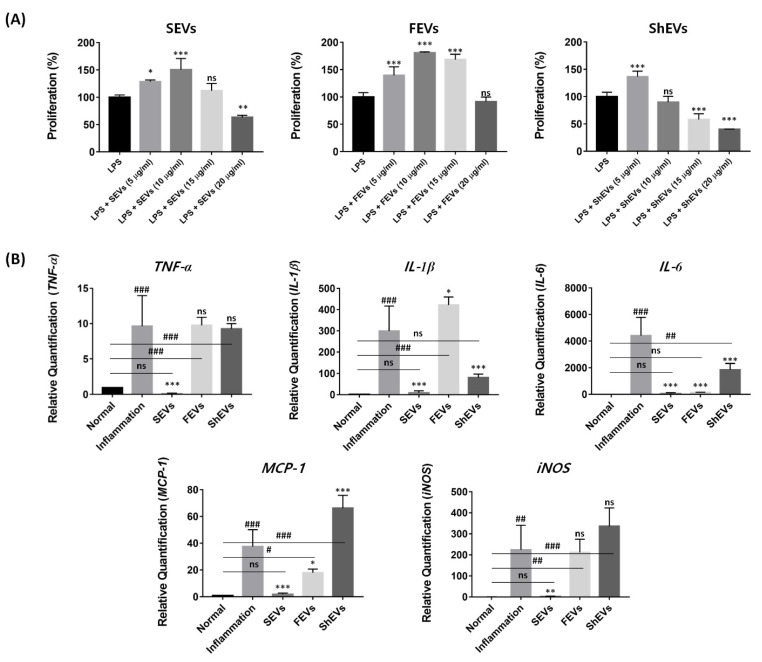
Marine organism ECM-anchored EVs have biological activity. (**A**) The effect of cell proliferation in inflammation environment was measured using water-soluble tetrazolium salt. The mEVs of several concentrations (0, 5, 10, 15, 20 μg/mL) were used to treat LPS (50 ng/mL)-induced inflammatory model for 24 h. Data are presented as the mean ± SD from independent experiments (*n* = 4). (* *p* < 0.001, ** *p* < 0.01, and *** *p* < 0.001, versus inflammation (*) calculated using one-way ANOVA) (**B**) Anti-inflammation activity of mEVs in LPS-induced model, pro-inflammatory cytokine mRNA (*TNF-α*, *IL-1β*, *IL-6*, *MCP-1*, and *iNOS*) expression was analyzed using RT-qPCR and normalized by using the *GAPDH* endogenous expression. Data are presented as the mean ± SD from independent experiments (*n* = 4). (*, # *p* < 0.001, **, ## *p* < 0.01 and ***, ### *p* < 0.001, versus inflammation (*) or normal groups (#) calculated using one-way ANOVA).

To investigate the effect of the interaction between inflammation and mEVs, we compared the cell proliferation rate and mRNA expression levels of cytokines and chemokines in macrophages that are important for innate immunity and regeneration [39,40]. For the cell proliferation assay, a lipopolysaccharide (LPS)-induced inflammatory in vitro model was treated with mEVs at 0, 5, 10, 15, and 20 μg/mL concentrations. In the SEV group, the highest proliferation increase (150%) was observed at 10 μg/mL; in contrast, the growth rate decreased at high concentrations of 15 and 20 μg/mL, and viability decreased to 63% at 20 μg/mL. In the FEVs group, in contrast to the normal environment, in the inflammation environment, a significant increase of up to 173% was observed at 10 μg/mL, followed by an 81% decrease at the highest concentration of 20 μg/mL. There was no significant increase in proliferation in the ShEV group (increase to 136% at 5 μg/mL). In addition, it was confirmed that viability decreased at other concentrations (Figure 5A).

Further, the expression of inflammatory chemokine and cytokine genes was compared. The mEV concentrations, when measuring the highest proliferation rate (SEVs: 10 μg/mL, FEVs: 10 μg/mL, and ShEVs: 5 μg/mL), were chosen and treated for 48 h. The relative quantification of messenger RNA (mRNA) of tumor necrosis factor-α (*TNF-α*), interleukin-1β (*IL-1β*), *IL-6*, macrophage chemoattractant protein-1 (*MCP-1*), and inducible nitric oxide synthase (*iNOS*) was performed (Table 1).

Real-time quantitative polymerase chain reaction (RT-qPCR) results showed that SEVs have powerful suppression effects. In the SEV group, the amount of *TNF-α* mRNA was smaller than that in the normal state, and *IL-1β*, *IL-6*, *MCP-1*, and *iNOS* were significantly decreased in the inflammation group. FEVs can suppress only *IL-6* mRNA synthesis; in contrast, *IL-1β* mRNA gene levels were increased. In addition, there were no statistical differences in *TNF-α*, *MCP-1*, and *iNOS* mRNA levels. The ShEV results showed that only the *IL-1β* mRNA gene could be suppressed, while the *MCP-1* gene level increased. The other cytokines showed no significant changes (Figure 5B).

These results may have occurred in differences in origin tissue. The composition of the ECM and the types of cells present in each tissue are also different. For example, factors involved in anti-angiogenesis and adhesion, which are rare in the cartilage-derived ECM [41], exist in other tissues, whereas factors involved in angiogenesis and adhesion are abundant in the uterine ECM [42,43]. As in the examples, there are differences in components and biological effects, because cells in each source tissue release ECMs to the outside to fit the environment [44]. Therefore, we assumed that differences in biological effects have depended on the ECM released according to the marine organism type and the environment facing each cell.

Among the three marine organisms’ EVs, the anti-inflammatory effect of SEVs was most clear. Previous studies with sea cucumber have also described the suppression of inflammatory environments. These studies reported that sea cucumber collagen and proteoglycan components ameliorate the inflammatory environment [45,46,47]. However, this study suggests the possibility that inflammation could be regulated by ECM-anchored EVs rather than by the effects of collagen or proteoglycan, which are primarily structural molecules.

## 3. Materials and Methods

### 3.1. Preparation of Marine EVs

Marine EVs were isolated using previously described protocols [26], with slight modifications. Briefly, sea cucumber (*Stichopus japonicus*) intestines were removed and stored in a freezer. Skin- and bone-removed flat fish (*Paralichthys olivaceus*) meat and shell-removed shrimp (*Litopenaeus vannamei*) bodies were collected and kept in a freezer. The frozen marine organisms were cut to a size of 1–2 cm and then agitated in a hypotonic buffer at 4 °C for 4 days, followed by centrifugation at 8000× *g* for 60 min at 4 °C to collect homogenized ECM. Lyophilized marine ECMs were treated with pepsin (Sigma-Aldrich, St. Louis, MO, USA) solution in HCl for 24 h at room temperature (RT). Digested ECMs were then neutralized to pH 7 using NaOH and dialyzed for 24 h at 4 °C. The desalted ECM suspensions were then lyophilized. Matrix-bound EVs are tightly anchored. To lyse marine ECM, lyophilized ECMs were treated with a collagenase (Worthington, OH, USA) solution in phosphate buffered saline (PBS) for 2 days at RT. Enzymatically digested ECM solution was spun down to remove the non-soluble part, followed by filtration with a 0.45 and 0.2 μm filter. The filtered solution was concentrated using a 100 kDa filter and treated with ExoQuick-TC (System Biosciences, Palo Alto, CA, USA) precipitation solution according to the manufacturer’s protocol.

### 3.2. Marine EV Morphology

Marine organism-derived EVs were visualized using TEM (H-7500; Hitachi, Tokyo, Japan) and SEM (MIRA 3 LMH; TESCAN, Brno, Czech Republic). Electron microscopy analysis was performed according to a previously described protocol. The mEV suspension (100 μg) was fixed with 4% paraformaldehyde for 30 min and washed with PBS. Subsequently, fixed EVs were dehydrated with an ascending sequence of ethanol (80%, 90%, 95%, and 100%). The mEV suspension was dropped on a cover glass or carbon-coated grid (TED PELLA, Redding, CA, USA) and left in fume hoods for 24 h.

### 3.3. Particle Size Analysis

The marine EVs were diluted in 0.2 μm filtrated PBS and determined using dynamic light scattering (DLS). Briefly, isolated marine EVs were diluted 1:200 to a final volume of 2000 μL and then placed in an optical grade cuvette. Size measurements were conducted at 25 °C using a size analyzer (Litsizer 500; Anton Paar, Graz, Austria) according to the manufacturer’s instructions. The data from three independent experiments were presented.

### 3.4. Cell Culture

RAW 264.7 cells (No. 40071; KCLB, Seoul, Korea) were grown in RPMI-1640 (GIBCO, Grand Island, NY, USA) supplemented with 10% heat-inactivated fetal bovine serum (FBS, GIBCO) and antibiotics (AA, GIBCO) at 37 °C under 5% CO_2_. The culture medium was replaced with fresh medium every 2–3 days. A cell scraper was used to harvest cells and protect the phenotypic changes.

### 3.5. Immune Modulation Assay

To test the anti-inflammatory effects of mEVs, RAW-264.7 cells were seeded in 24-well plates. After 24 h, cells were treated with each marine EV suspension (SEVs, 10 μg/mL; FEVs, 10 μg/mL; ShEVs, 5 μg/mL) in FBS-free RPMI-1640 medium with LPS (50 ng/mL) (Sigma-Aldrich) for 48 h. Messenger RNA (mRNA) was collected after 2 days for RT-qPCR analysis.

### 3.6. Fluorescence Microscopy

To track the marine EVs, the surface membrane was labeled with PKH-26 red fluorescent cell linker (Sigma-Aldrich) for 24 h at 4 °C. Subsequently, the remaining PKH-26 dye was washed three times using PBS and 100 kDa filter and diluted in serum-free RPMI-1640 containing AA to treat RAW-264.7 cells for 12 h at 37 °C under 5% CO_2_. After 12 h, the sample medium was changed to DAPI fluorescent dye (Invitrogen, Waltham, MA, USA) for 15 min and then washed twice with PBS. Endocytosis was observed using a fluorescence microscope (Axio-Observer 5; Carl Zeiss, Oberkochen, Germany).

### 3.7. Protein Cargo Analysis

To determine the protein concentration and size distribution of marine EV cargo, Bradford assay and SDS-PAGE were performed. Briefly, marine EVs and membrane lysis buffer were mixed in a 1:1 ratio. Protein concentrations were measured using the Bradford assay (Bio-Rad, Hercules, CA, USA).

The destroyed EVs were diluted at a 1:1 ratio with a 2X Laemmli sample buffer (Bio-Rad) containing 5% beta-mercaptoethanol (Sigma-Aldrich) and then boiled at 95 °C for 5 min. Each sample (20 μg) was run on 4%–20% precast SDS-PAGE gels (Bio-Rad). Then, the gel was washed twice with distilled water and treated with Coomassie Brilliant Blue (Bio-Rad) for 30 min at RT, washed with distilled water, and left for destaining overnight. The gel was visualized using gel-doc system (280; Azure Biosystems, CA, USA).

### 3.8. Gene Cargo and Gene Expression Analysis

To verify the genetic material of marine EV cargo, the genetic material was extracted using TRIzol (Invitrogen) and chloroform (Sigma-Aldrich). Total RNA quantity was measured using a SpectraDrop Micro-Volume Microplate (Molecular Devices, San Jose, CA, USA).

LPS-induced inflammatory model-derived mRNA was extracted using an isolation kit (Bioneer, Daejeon, Korea), transcribed using a cDNA synthesis kit (Cellsafe, Yongin, Korea), and then kept at −20 °C. RT-qPCR was performed using pro-inflammatory cytokine primers and 2X SYBR Green Reaction Mix (Thermo Fisher Scientific, Waltham, MA, USA) according to the manufacturer’s protocol. The relative gene expression levels of the samples were normalized to glyceraldehyde 3-phosphate dehydrogenase (*GAPDH*) as an internal control.

### 3.9. Assessment of Cell Viability and Proliferation

The effect of marine EVs on proliferation in normal or inflammatory environments was evaluated using the water-soluble tetrazolium salt (WST) assay (EZ-cytox, DoGenBio, Seoul, Korea). Briefly, based on the protein concentration measured by the Bradford assay, mEVs were diluted to 5, 10, 15, and 20 μg/mL in FBS-free RPMI-1640 medium and treated onto RAW 264.7 cells for 24 h. In the inflammatory environment, the same concentration of LPS (50 ng/mL) was used. The WST assay was performed according to the manufacturer’s instructions.

To check the cell viability, highest concentration marine EVs (20 μg/mL) in FBS-free medium were treated on RAW-264.7 for 24 h. Then, the medium was changed using the live and dead assay kit containing calcein-AM and ethidium homodimer (Invitrogen) in Hanks’ balanced salt solution (HBSS, Welgene, Gyeongsan, Korea) for 1 h. After 1 h, live and dead cells were observed using a fluorescence microscope (Carl Zeiss).

### 3.10. Statistical Analysis

GraphPad Prism version 7.0 (GraphPad, San Diego, CA, USA) was used to produce graphic images and perform statistical analysis. Data are expressed as mean ± standard deviation (SD) from at least three independent experiments. Statistical significance was analyzed by using Student’s t-test and one-way analysis of variance (ANOVA), followed by a Tukey–Kramer post hoc test. A value of *p* < 0.05 was considered as statistically significant (*, # *p* < 0.05, **, ## *p* < 0.01, and ***, ### *p* < 0.001).

## 4. Conclusions

In the field of regenerative medicine, the world’s most trending fields are exosomes and EVs [48,49]. Treatment using cells is difficult to obtain approval by FDA regulations, and biomaterials such as the ECM, alginate [50], and hydroxyapatite [51] are used for tissue repair and replacement, but their effectiveness as a treatment is insufficient. On the other hand, EVs are positioned as an important factor in the field of regenerative medicine, are easy to apply regulations to [52], and have effects corresponding to cells [53,54]. However, despite research on various marine materials, no studies have been reported on expanding EVs, which are attracting the most attention, to marine organisms. Characterization and biological activation studies of marine organism ECM-anchored EVs (mEVs) were conducted in this study. We successfully established protocols for extracting EVs from the ECM of representative marine organisms, such as echinoderms, fish, and crustaceans. The morphological characteristics of mEVs were found to be similar to those of mammalian and cell-derived EVs. In addition, it was confirmed that it has a lipid membrane structure that can protect internal elements, such as proteins and genetic materials, during decellularization. From the results of the cell activity test, we confirmed that mEVs were not toxic. Extracted mEVs have been shown to suppress LPS-induced expression of inflammatory cytokines and chemokines in macrophages. The most dramatic decrease was observed in sea cucumber ECM-anchored EVs (SEVs). We expected that the cargo inside the SEVs would inhibit inflammatory cytokine gene levels by acting on the immune mechanism of macrophages. Although the research on mEVs is still in its infancy, this study is expected to attract attention toward mEVs as a new factor that can be used in regenerative medicine and immunotherapy. Furthermore, we suggested the possibility of expanding the experiment to various types of marine animals and inedible marine animals, such as star fish and jelly fish, and marine waste, such as fish skin and urchin body wall from which the ECM can be extracted. In particular, we believe that EVs derived from echinoderms deserve attention. If the regenerative and physiological abilities of echinoderms [55,56] can be mimicked using echinoderm ECM-anchored EVs, it is expected to become an important element among marine drugs and high value-added therapeutics to be studied in the future.

## Figures and Tables

**Table 1:** Primer sequences for real-time polymerase chain reaction.

Target	Forward Sequences (5′–3′)	Reverse Sequences (5′–3′)
*TNF-α*	TTCTCATTCCTGCTTGTGGC	GGGAACTTCTCATCCCTTTGG
*IL-1β*	AAAGCTCTCCACCTCAATGG	GCCGTCTTTCATTACACAGG
*IL-6*	GATACCACTCCCAACAGACC	GCAAGTGCATCATCGTTGTTC
*MCP-1*	CCTGCTGCTACTCATTCACC	CTGGACCCATTCCTTCTTGG
*iNOS*	GTGGTGACAAGCACATTTGG	GAACTGAGGGTACATGCTGG
*GAPDH*	TTCAACAGCAACTCCCACTC	TCCTTGGAGGCCATGTAGG
